# Mental health burden associated with specific ocular conditions among Medicare Advantage beneficiaries with type 2 diabetes: examining the impact of glaucoma, cataracts, retinopathy, and dry eye on unhealthy mental health days

**DOI:** 10.3389/fpubh.2026.1708485

**Published:** 2026-04-16

**Authors:** Michael D. Twa, Tonghui Xu, Patrick Dang, Lucy Kehinde-Darnell, Jessica Dobbins, Winston Liaw, Omolola E. Adepoju

**Affiliations:** 1 College of Optometry, University of Houston, Houston, TX, United States; 2 College of Medicine, University of Houston, Houston, TX, United States; 3 Humana Inc., Louisville, KY, United States

**Keywords:** Medicare Advantage beneficiaries, mental health, ocular conditions, social detereminants, type 2 dabetes mellitus

## Abstract

**Objective:**

To examine differences in mental health burden across ocular conditions among Medicare Advantage patients with type 2 diabetes in the United States.

**Methods:**

Pooled national claims data were obtained from 992 individuals with type 2 diabetes and ocular conditions who completed the CDC Health-Related Quality of Life questionnaire between 2016 and 2020. Covariates included demographic characteristics and health conditions. A generalized linear regression model (GLM) was used to examine the association between ocular conditions and unhealthy mental health days. Stratified analyses were conducted for patients with and without dry eye disease (DED). Average marginal effects (AMEs) and adjusted predicted means were additionally calculated to aid interpretation.

**Results:**

Among these patients, 42% reported at least 1 day of poor mental health, and 28% had DED. The GLM indicated that patients with glaucoma only experienced 2.69 times more unhealthy mental health days (95% CI: 1.85–3.90). Within the DED subgroup, patients with glaucoma only experienced 7.80 times more unhealthy mental health days (95% CI: 4.61–13.88). No other ocular condition was significantly associated with unhealthy mental health days.

**Conclusion:**

Mental health burdens are associated with ocular conditions among Medicare Advantage patients with type 2 diabetes, underscoring the need for targeted interventions to address social risk factors in this population.

## 1 Introduction

Diabetic retinopathy, a microvascular disorder damaging retinal blood vessels arising from long-term diabetes mellitus (1), is the primary cause of vision impairment among working age individuals (2). The prevalence of type 2 diabetes mellitus is concurrently rising with diabetic retinopathy, effecting an estimated 32 million adults (3) and 9.6 million adults (4) in the U.S. respectively, raising significant public health concern. Leading organizations, including the American Diabetes Association and the American Optometric Association, have highlighted this growing burden and its implications for visual function. Left undiagnosed and untreated, diabetic eye disease and other conditions often lead to vision impairment and blindness, which result in difficulties with activities of daily living, reduced quality of life, and mental distress (5).

The dual burden of ocular conditions and diabetes is especially pronounced among older adults, approximately one in three of whom live with diabetes (6). Age-related ocular disorders, such as glaucoma (7) and cataracts (8) are highly prevalent in this population, often compounding the physical and emotional toll of chronic diseases such as diabetes (9). This intersection becomes even more concerning when layered with the high prevalence of mental health challenges in older adults, including social isolation, depression, and loneliness (10). The convergence of visual impairment, chronic illness, and psychological distress can severely compromise quality of life, impede effective self-management, and elevate the risk of adverse health outcomes. Addressing this complex scenario requires an integrated, person-centered approach that simultaneously addresses the physical and mental health needs of older adults.

Previous studies have documented associations between mental health and diabetic complications (11). For example, Individuals with type 2 diabetes who experience diabetic retinopathy (e.g., diabetic macular edema, or retinal vasculopathy) were found to be at increased risk for depression and anxiety, which in turn is associated with lower treatment adherence and a greater likelihood of developing further diabetes-related complications over time (12). Similarly, studies have documented associations between ocular conditions and mental distress. Work by Zhang et al. (13) found an association between cataract status and depression, following adjustments for variables such as age, gender, educational level, income, and visual acuity. In patients with glaucoma, control of intraocular pressure (IOP) and measures of disease progression have been associated with an increased risk of anxiety (14). Dry eye disease (DED), which is characterized by ocular discomfort (dryness, foreign body sensation, etc.) infections, and poor vision that can affect quality of life and daily activities (15). This condition and its severity, is positively associated with an increased prevalence of depression and anxiety (16).

Prior studies have proposed mechanisms linking ocular conditions to mental health through the functional and psychosocial consequences of vision loss (17). Impaired vision can reduce the ability to perform daily activities, increase dependence on others, limit mobility (18), and contribute to social isolation (19). Fear of disease progression and the burden of managing comorbidities, including type 2 diabetes, may further heighten psychological distress.

While prior literature has documented the mental health burden associated with both diabetes and ocular conditions independently, few studies have examined their combined impact. Existing research rarely considers multiple ocular comorbidities simultaneously or focuses specifically on older adults, who bear a disproportionate burden of both vision impairment and mental health disorders. Our study addresses this gap by evaluating how co-occurring ocular conditions relate to unhealthy mental health days among older adults with type 2 diabetes. The co-occurrence of these conditions—particularly among older adults—represents an underexplored yet important area of study with potential implications for integrated care and health policy. As measured by the Centers for Disease Control and Prevention (CDC) Health-Related Quality of Life (HRQOL) tool (20), the “unhealthy mental health days” metric serves as a valuable population-level indicator of psychological distress. Its correlation with vision loss underscores the broader impact of ocular conditions on mental well-being and highlights the need for integrated interventions.

This study leverages health insurance claims data from Medicare Advantage (MA) beneficiaries with type 2 diabetes to quantify the burden of unhealthy mental health days within this vulnerable population. To test the hypothesis that the association between ocular conditions (i.e., glaucoma, cataracts, and retinopathy) and mental health might be stronger among patients who also experience DED symptoms, we stratify our analyses by the presence or absence of DED, a condition frequently associated with both diabetes and psychological distress. Findings from this work aim to inform policy and clinical practice by supporting the development of comprehensive, ocular health and vision-inclusive strategies for mental health and diabetes management. Ultimately, studies of this nature may contribute to mitigating the progression of these interconnected conditions and enhancing quality of life for older adults living with type 2 diabetes.

## 2 Method

### 2.1 Data

**Figure 1:**
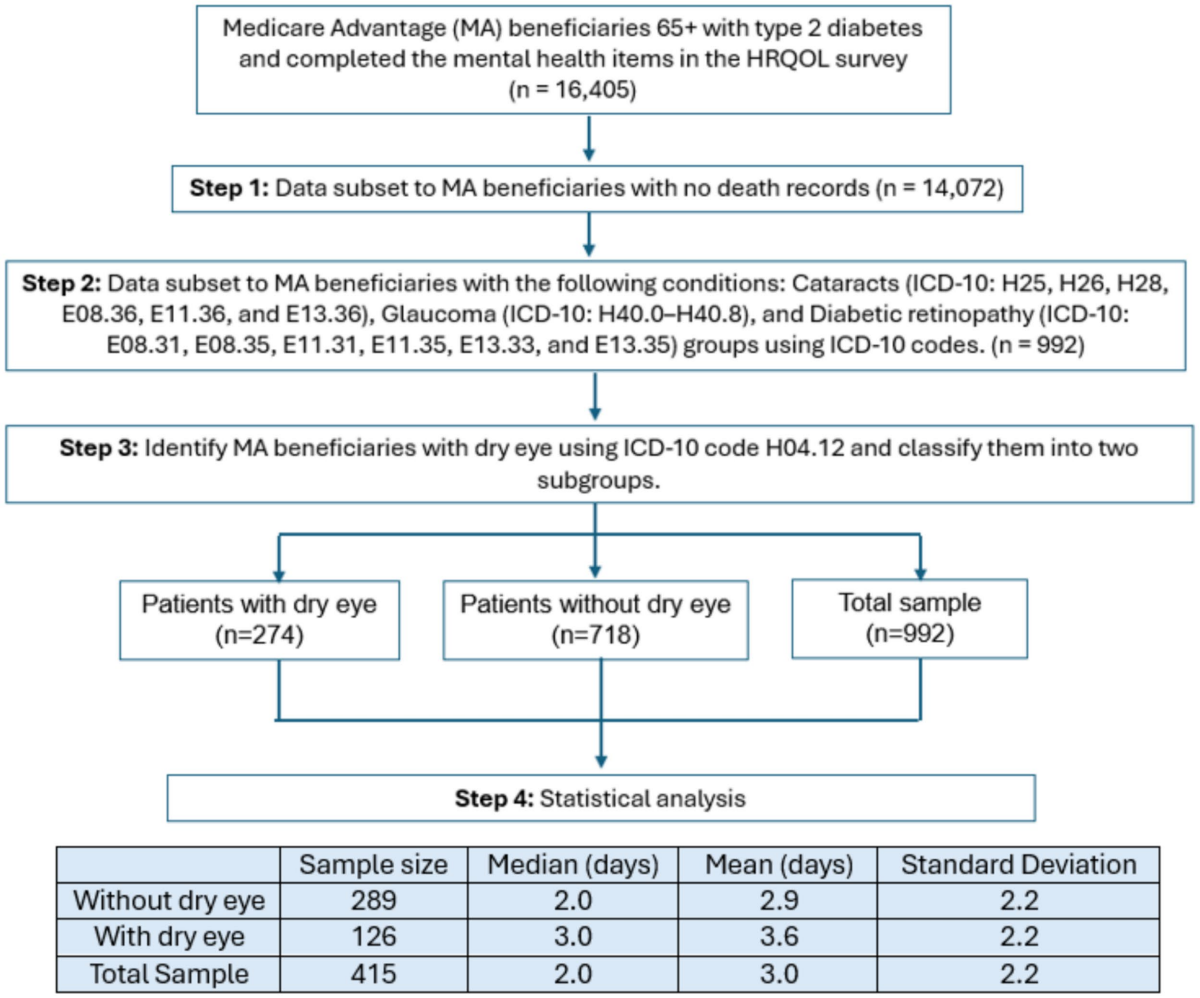
Analytic cohort selection from claims data.

Cross-sectional data were obtained from Humana, Inc., a U.S. based insurance company, and included MA subjects aged 65 and older who had diabetes had completed the CDC HRQOL questionnaire in 2019 and 2020. Figure 1 shows the analytic cohort selection from claims data. First, we identified patients who completed the HRQOL survey and had type 2 diabetes. We then identified individuals with glaucoma (ICD-10: H40.0–H40.8), diabetic retinopathy (ICD-10: E08.31, E08.35, E11.31, E11.35, E13.33, and E13.35), cataracts (ICD-10: H25, H26, H28, E08.36, E11.36, and E13.36), or any combination of these conditions using their respective ICD-10 codes. Next, we included DED (ICD-10: H0412) as a control variable to stratify records into two groups: a) those without DED at index and b) those with DED. Patients without HRQOL data, in MA plans contractually excluded from research, or those who died, were excluded from this study.

### 2.2 Measures

The continuous outcome variable, unhealthy mental health days, was derived from respondents’ self-reported number of days in the past 30 days when they experienced poor mental health (0–30 days). The independent variables included demographic characteristics such as biological sex (female and male); age, categorized into two groups: 65–74, and 75+; race/ethnicity (Non-Hispanic White and minority groups); primary language (English and Spanish); education level (less than high school, high school, and above high school); annual adult income (≤ 25,000 USD and > 25,000 USD); marital status (married and single or others); and dual status (indicating whether individuals are enrolled in both Medicaid and Medicare or not). Other independent variables included whether the patient currently lives in a rural area, the Body mass index (BMI) category (i.e., 25 kg/m^2^ vs. 25 kg/m^2^ or higher), and their Diabetes Complications Severity Index (DCSI) score, which is a 13-point scale that combines the number and severity of complications.

### 2.3 Missing data imputation

Primary language, education level, adult annual income, marital status, and race/ethnicity had missing data rates of 26.2, 13.9, 13.9, 20.1, and 4.6%, respectively. During the data cleaning process, observations with missing outcome variables were removed using listwise deletion. First, we set *m* = 0 to examine the missing-data pattern. We then addressed missing data under a missing-at-random (MAR) assumption using multiple imputation (*m* = 10) with the fully conditional specification (FCS) logistic regression method (LINK = GLOGIT) in SAS PROC MI, with 50 iterations per imputation. The imputation model included all analysis variables with and without missing data (including exposures, covariates, and outcomes) as predictors to preserve their multivariate relationships. Among these variables, only the DCSI score and the outcome were continuous; all other variables were treated as categorical. Missing values in categorical variables were imputed using the FCS LOGISTIC statement. To assess the reliability of the imputations, we evaluated the “Logistic Models for FCS Method” output to confirm that effect estimates were stable across imputation rounds. Parameter estimates and standard errors across the 10 imputed datasets were pooled using Rubin’s rules via PROC MIANALYZE. We also confirmed that the relative efficiency for all parameter estimates was high (≥ 0.95), indicating that 10 imputations were sufficient. For sensitivity analysis, we conducted a complete-case analysis. A comparison of effect estimates from the complete-case and imputed analyses is presented in Supplementary Table A1.

### 2.4 Analysis

After data cleaning, the final sample size was 992, with no missing data. Descriptive analyses, including frequencies, proportions, and means, were performed to summarize participant demographic and clinical characteristics. Generalized linear models (GLM) with a log link and Tweedie distribution were fitted independently to three cohorts a) the total sample, b) the subgroup of primary interest: participants with DED, and c) the subgroup of participants without DED, which served as a comparative group. The Tweedie distribution was selected based on the observed outcome distribution (substantial right skewness with a large proportion of zeros), goodness-of-fit criteria, and model convergence, and because unhealthy mental health days reflect aggregated symptom burden over a fixed 30-day recall period rather than counts of independent events. To determine potential residual confounding by socioeconomic factors in the rural–urban association, we conducted sensitivity analyses comparing two model specifications. Because a log link was used in the model, we applied the exponential function to convert the log-transformed beta coefficients. To statistically evaluate whether DED status significantly modifies the associations of key covariates, we fit additional GLMs to the total sample. We ran five additional models, each including an interaction term between DED status and one of the following candidate modifiers: gender, marital status, education level, dual eligibility status, and rural residence, to examine whether DED status modified these associations. Additionally, average marginal effects (AMEs) were computed for all three models on the original outcome scale to facilitate interpretation, and adjusted predicted means were estimated for ocular condition variables with statistically significant associations to further quantify the magnitude of these effects. All analyses were conducted in SAS 9.4, with findings considered significant at *p* < 0.05. Exponential coefficients, standard errors, 95% confidence intervals (CIs), goodness-of-fit statistics (e.g., scaled Pearson chi-square, dispersion, and power parameters), and multicollinearity diagnostics (e.g., variance inflation factors) were computed. The scaled Pearson chi-square statistic, dispersion parameter, and power parameter were estimated from the data via maximum likelihood.

## 3 Results

### 3.1 Statistical descriptive

Table 1 shows the characteristics of study participants. 54% of the sample had cataracts only, 27% had cataracts and glaucoma, 11% had glaucoma only. The others category (*n* = 78; 8%) included patients with multiple ocular conditions. Among these patients, 11 had glaucoma and retinopathy (1.0%), 49 had cataract and retinopathy (5.0%), 5 had retinopathy only (0.6%), and 13 had all three conditions (1.4%). Overall, 55% of the patients were female and 45% were male. With regards to age, 47% were between 65 and 75 years old. Approximately 86% lived in non-rural areas while 14% resided in rural areas. Among these patients, 51% had an annual income of 25,000 USD or less. Additionally, 26% had some college experience, and 53% were married. The majority (94%) spoke English, while 9% spoke Spanish. Approximately 72% identified as non-Hispanic White, while 28% belonged to minority groups, including Black or African American, Hispanic, Asian, or other backgrounds. About 10% had eligible dual status, and with regards to BMI, only 2.5% had a BMI below 25 kg/m^2^, 66.73% had a BMI between 25 kg/m^2^ and 30 kg/m^2^. Mean DCSI score was 2.6. Overall, 72% had no DED.

**Table 1:** Characteristics of study sample and adjusted pooled models examining association with unhealthy mental health days.

	Freq (%)	Exp (*β*)	95% CIs
Ocular condition			
Glaucoma only	110 (11.09)	2.69 (0.19)***	[1.85, 3.90]
Cataracts only	531 (53.53)	REF	[0.64, 1.12]
Cataracts with glaucoma	273 (27.52)	0.85 (0.14)	[0.71, 1.62]
Others^1^	78 (7.86)	1.07 (0.21)	
Gender			
Male	447 (45.06)	0.90 (0.12)	[0.72, 1.15]
Female	545 (54.94)	REF	
Age			
65–75	469 (47.28)	REF	[0.73, 1.13]
75+	523 (52.72)	0.90 (0.11)	
Currently living in a rural area			
No	851 (85.79)	REF	[0.41, 0.82]
Yes	141 (14.21)	0.58 (0.18)*	
Annual adult income			
≤ 25,000 USD	513 (51.71)	1.61 (0.16)**	[1.16, 2.24]
> 25,000 USD	479 (48.29)	REF	
Education			
Less than high school	356 (35.89)	REF	[0.59, 1.13]
High school	376 (37.90)	0.81 (0.16)	[0.49, 1.48]
Above high school	258 (26.11)	0.85 (0.27)	
Marital status			
Married	523 (52.72)		
Single/others	468 (47.28)	1.89 (0.13)***	[1.45, 2.46]
Primary Language			
English	926 (93.45)	REF	
Spanish	65 (6.55)	1.51 (0.21)*	[1.09, 2.52]
BMI			
Below 25 kg/m^2^	24 (2.42)	0.58 (0.13)	
25–30 kg/m^2^	662 (66.73)	1.20 (0.12)	[0.33, 1.53]
Over 30 kg/m^2^	306 (30.85)	REF	[0.93, 1.54]
Race/ethnicity			
Non-Hispanic White	714 (71.98)	REF	
Other races	278 (28.02)	1.47 (0.12)**	[1.67, 1.84]
Dual eligibility status			
No	887 (89.42)		
Yes	105 (10.58)	0.91 (0.17) REF	[0.66, 1.27]
DED status			
No	718 (72.38)	REF	
Yes	274 (27.62)	1.24 (0.11)	[0.99, 1.55]
DCSI [Mean (SD)]	2.56 (±2.09)	1.11 (0.03)***	[1.06, 1.17]
Total sample size	992	—	—

*p < 0.05; **p < 0.01; ***p < 0.001. DCSI, Diabetes Complications Severity Index; SD, standard deviation. ^1^combined because of small cell sizes for each category. REF, reference group. The value inside the parentheses is the standard error. Others include retinopathy only (*n* = 5; 0.6%), Glaucoma with Retinopathy (*n* = 11; 1%)/cataracts with retinopathy (*n* = 49; 5%)/all three conditions (*n* = 13; 1.4%). Other races include Black or African American/Hispanic/Asian/others.

Figure 1 also presents the descriptive statistics of patients reporting unhealthy mental health days. Overall, 415 (41.8%) reported at least one unhealthy mental health day; however, in the subgroup without DED, 289 (40.3%) reported at least one unhealthy mental health day compared to 126 (46.0%) in the subgroup with DED. The average number of unhealthy mental health days was 3.0 in the total sample, 2.9 in the DED group and 3.6 days in the group without DED.

### 3.2 Sensitivity analysis for the missing data imputation and model fits

We conducted sensitivity analyses by comparing two approaches: a primary FCS imputation model and a complete-case analysis. Both approaches achieved convergence, and RE values were acceptable. Supplementary Table A1 presents the results of the sensitivity analysis using complete-case analysis, including the sample size and the corresponding GLM model estimates. Approximately 42% of the data were removed in the complete-case analysis; as a result, the total sample size was 576. Compared to complete-case analysis and primary model, although the complete-case analysis suggested that nearly all variables were statistically significant, the multiple imputation analysis identified a smaller set of significant predictors. This pattern suggests that some associations observed in the complete-case analysis may be sensitive to missing data handling. We therefore focus on the imputed results as the primary analysis. For the exposure, we observed changes in the statistical significance: Glaucoma only in the oracular condition [Exp (*β*) = 2.69, 95% CI: 1.85–3.90 vs. = 2.32, 95% CI: 1.58–3.39] and Cataracts with Glaucoma in the ocular condition [Exp (*β*) = 0.85, 95% CI: 0.64–1.12 vs. Exp (*β*) = 0.17, 95% CI: 0.07–0.42]. DED condition [Exp (*β*) = 1.24, 95% CI: 0.99–1.55 vs. Exp (*β*) = 2.63, 95% CI: 1.94–3.42]. This pattern suggests that the point estimates of the effects of DED and ocular conditions on mental health may be influenced by the complexity of missing data preprocessing methods. In particular, the use of list wise deletion, which removed approximately 42% of the original sample, may have contributed to potential bias in the estimates.

In addition, we evaluated multicollinearity using variance inflation factors. No variables had VIF values greater than 5, indicating a low risk of multicollinearity in all models. Goodness-of-fit statistics for all models are presented in Supplementary Table A4 (Part 3), and all models showed acceptable fit. The scaled Pearson Chi-squared ranged from 0.78 to 1.28 across models, indicating appropriate variance specification as values close to 1 suggest minimal over or under-dispersion. The dispersion parameters ranged from 1.39 to 2.44, reflecting moderate variability in the outcome beyond what would be expected under a Poisson distribution. The estimated power parameters ranged from 1.10 to 1.20, indicating a distributional form intermediate between Poisson and gamma, which supports the use of a compound Poisson–gamma model for outcomes characterized by a substantial proportion of exact zeros. Model diagnostics confirmed that the Tweedie distribution was an appropriate choice for GLMs.

### 3.3 GLM regression

Table 1 depicts the adjusted models examining the associations between ocular conditions and unhealthy mental health days. Our results indicate that compared to patients with cataracts only, patients with glaucoma only had 2.69 times more unhealthy mental health days (95% CI: 1.85, 3.90). Conversely, rural residents had 42% fewer unhealthy mental health days compared to urban residents [Exp (*β*) = 0.58, 95% CI: 0.41–0.82]. Additionally, patients with an annual income below or equal to $25,000 experienced 1.61 times more unhealthy mental health days (95% CI: 1.16–2.24). Patients who were single or had other marital statuses 1.89 times more unhealthy mental health days (95% CI: 1.45–2.46). Patients whose primary language was not English experienced 1.51 times as many unhealthy mental health days (95% CI: 1.09–2.52). Compared to non-Hispanic White patients, individuals from minority groups reported 1.47 times as many unhealthy mental health days (95% CI: 1.67–1.84). Patients with higher DCSI scores [Exp (*β*) = 1.11, 95% CI: 1.06–1.17] reported 11% more unhealthy mental health days. DED was not statistically significant in the primary model. In addition, to assess potential residual confounding by socioeconomic factors in the rural–urban association, we conducted sensitivity analyses comparing two model specifications (see Supplementary Table A3). The comparison revealed that the rural residence remained robust [Exp (*β*) = 0.58, 95% CI: 0.41–0.82 vs. Exp (*β*) = 0.67, 95% CI: 0.48–0.93]. Notably, while the effect estimates for some covariates were attenuated in the fully adjusted model, the association between rural residence and fewer unhealthy mental health days persisted.

Table 2 depicts the adjusted stratified model by DED status. For those without DED, DCSI score was significantly associated with prolonged unhealthy mental health days. Female patients had 36% fewer expected unhealthy mental health days compared with male patients [Exp (*β*) = 0.64, 95% CI: 0.46–0.89]. Rural residents had 50% fewer unhealthy mental health days compared to urban residents [Exp (*β*) = 0.50, 95% CI: 0.32–0.79]. Patients with single or other marital status reported 2.08 times as many unhealthy mental health days [Exp (*β*) = 2.08; 95% CI: 1.54–2.80]. Compared with participants who had less than a high school education, those with more than a high school degree [Exp (*β*) = 0.49, 95% CI: 0.29–0.84] had 36 and 58% fewer expected unhealthy mental health days, respectively. Patients with higher DCSI scores [Exp (*β*) = 1.21, 95% CI: 1.14–1.52] reported 20% more unhealthy mental health days. Patients without dual insurance status had 37% fewer expected unhealthy mental health days compared with male patients [Exp (*β*) = 0.64, 95% CI: 0.46–0.89].

**Table 2:** Adjusted pooled stratified models by DED status.

	Model 2: patients without DED (*n* = 718)			Model 2: patients with DED (*n* = 274)		
	Freq (%)	Exp (*β*)	95% CIs	Freq (%)	Exp (*β*)	95% CIs
Ocular condition						
Glaucoma only	61 (8.50)	1.05 (0.18)	[0.43, 2.58]	49 (17.88)	7.80 (0.28)***	[4.61, 13.88]
Cataracts only	404 (56.27)	REF	[0.58, 1.16]	127 (46.35)	REF	[0.16, 1.75]
Cataracts with glaucoma	175 (24.37)	0.82 (0.25)	[0.50, 1.33]	98 (35.77)	0.52 (0.61)	
Others	78(10.86)	0.82 (0.46)		—	—	
Gender						
Female	342 (47.63)	0.64 (0.17)**	[0.46, 0.89]	203 (74.09)	3.61 (0.49)*	[1.30, 10.20]
Male	376 (52.37)	REF		71 (25.91)	REF	
Age						
65–74	371 (51.67)	REF	[0.90, 1.52]	27 (9.85)	REF	[0.38, 2.78]
75+	347 (48.33)	1.17 (0.13)		76 (27.74)	1.02 (0.48)	
Living in a rural area						
No	616 (85.79)	REF	[0.32, 0.79]	235 (85.77)	REF	[0.46, 7.24]
Yes	102 (14.21)	0.50 (0.23)**		39 (14.23)	1.83 (0.67)	
Primary language						
English	660 (91.92)	REF	[0.45, 1.46]	267(97.44)	REF	[1.14, 68.84]
Spanish	58 (8.02)	0.81 (0.30)		7 (2.56)	8.87 (0.97)*	
BMI						
Below 25 kg/m^2^	22 (3.06)	0.49 (0.56)	[0.16, 1.53]	2 (0.73)	0.84 (1.07)	[0.10, 6.92]
25–30 kg/m^2^	458 (63.79)	0.17 (0.18)	[0.94, 1.84]	204 (74.45)	0.66 (0.45)	[0.26, 1.70]
Over 30 kg/m^2^	238 (33.15)	REF		68 (24.82)	REF	
Annual adult income						
≤ 25,000 USD	379 (52.79)	1.13 (0.35)	[0.62, 2.78]	134 (48.91)	0.78 (0.78)	[0.13, 3.87]
> 25,000 USD	339 (47.21)	REF		140 (51.09)	REF	
Education						
Less than high school	270 (37.60)	REF	[0.29, 0.84]	86 (31.39)	REF	[0.60, 13.61]
High school	244 (33.98)	0.49 (0.26)*	[0.11, 1.81]	132 (48.18)	2.85 (0.73)	[0.14, 221.85]
Above high school	204 (28.42)	0.44 (0.64)		54 (19.71)	5.56 (1.67)	
Marital status						
Married	450 (62.67)	REF	[1.54, 2.80]	74 (37.01)	REF	[0.14, 1.22]
Single/others	268 (37.33)	2.08 (0.15)***		200 (72.99)	0.41 (0.51)	
Race/ethnicity						
Non-Hispanic White	491 (68,38)	REF	[0.77, 1.27]	223 (81.39)	REF	[8.96, 114.44]
Other races	227 (31.62)	0.99 (0.13)		51 (18.61)	32.03 (0.64)***	
Dual eligibility status						
No	631 (87.88)	0.64 (0.20)**	[0.46, 0.89]	256 (93.43)	4.69 (0.97)*	[1.54, 14.25]
Yes	87 (12.12)	REF		18 (6.57)	REF	
	Mean			Mean		
DCSI (continuous)	2.56 (2.05)	1.21 (0.03)***	[1.14, 1.52]	2.54 (2.18)	1.11 (0.02)	[0.88, 1.40]

*p < 0.05; **p < 0.01; ***p < 0.001. REF, reference group; DCSI, Diabetes Complications Severity Index; CIs, Confidence Intervals. The value inside the parentheses is the standard error. Others include Retinopathy only, Glaucoma with Retinopathy/Cataracts with Retinopathy/all three conditions. Other races include Black or African American/Hispanic/Asian/others.

Among participants with DED, those with glaucoma only had 7.80 times the expected number of unhealthy mental health days compared with patients with cataracts only (95% CI: 4.61–13.88). Female patients had 3.61 times the expected number of unhealthy mental health days compared with male patients (95% CI: 1.30–10.20). Patients whose primary language was not English had 8.87 times the expected number of unhealthy mental health days compared with cataracts-only patients (95% CI: 1.14–68.84). Minority group members had 32.03 times the expected number of unhealthy mental health days compared with non-Hispanic White individuals (95% CI: 8.96–114.44). Patients without dual insurance status had 4.69 times the expected number of unhealthy mental health days compared with those with dual insurance coverage (95% CI: 1.54–14.26). We observed several large Exp (*β*) estimates with wide 95% CIs in the DED model, particularly for language and race/ethnicity variables. For example, for minoritized racial groups, the outcome distribution within this group showed substantial heterogeneity: 53% of patients reported 5–7 unhealthy mental health days, whereas 47% reported zero unhealthy mental health days. This highly polarized, zero-inflated distribution likely contributed to both the large magnitude of the exponentiated coefficient and the wide CI. The maximum leverage value was 0.58, whereas the maximum Cook’s distance was 0.06. Although some observations exhibited relatively high leverage, the small Cook’s distance indicates that no individual observation exerted meaningful influence on the parameter estimates.

### 3.4 Interaction analyses, average marginal effects, and adjusted predicted means

We formally tested effect modification by including interaction terms between DED status and key covariates in a model fitted to the total sample. The results of these interaction tests are presented in Supplementary Table A3. DED status significantly modified the associations between rural residence, dual eligibility status, education level, gender, and unhealthy mental health days. In contrast, no statistically significant interactions were observed for marital status, indicating that the associations of this factor with unhealthy mental health days.

We also calculated AMEs for all three models, with results presented in Supplementary Table A4. In the total sample, participants with glaucoma only had an AME of 0.98 days (95% CI: 0.61–1.36), indicating that, on average, they experienced approximately one additional unhealthy mental health day over the 30-day period compared with participants with cataract only. Among participants with DED, the AME increased to 2.07 days (95% CI: 1.53–2.63). In addition, patients from minoritized racial groups had an AME of 3.47 days (95% CI: 2.19–4.74).

Because glaucoma was the only ocular condition showing a statistically significant association in the primary models, we further compared adjusted predicted mean unhealthy mental health days between the glaucoma-only and cataract-only groups (Supplementary Table A4, Part 2). In the total sample, the adjusted predicted mean was higher among glaucoma-only participants (0.74 days; 95% CI: 0.06–1.43) than among cataract-only participants (0.42 days; 95% CI: 0.04–0.80). Although this contrast reached statistical significance in the total sample, the absolute magnitudes of the adjusted predicted means for both groups were less than 1 day over the 30-day recall period, indicating a low overall burden of unhealthy mental health days.

Glaucoma-only was the only ocular condition showing a statistically significant association; therefore, to enhance interpretability for public health readers, we compared adjusted predicted means between the glaucoma-only and cataract-only groups in Supplementary Table A4 (Part 2). Among participants without DED, the adjusted predicted means for both glaucoma-only and cataract-only groups were close to zero, and no statistically significant differences were observed. In contrast, among participants with DED, adjusted predicted mean unhealthy mental health days were substantially higher for both ocular conditions, ranging approximately from 2 to 13 days per month. However, these estimates were not statistically significant and were accompanied by wide confidence intervals, reflecting considerable uncertainty. This lack of statistical significance is likely attributable, at least in part, to limited sample size and greater variability within the DED subgroup.

## 4 Discussion

In our study assessing the mental health burdens associated with ocular conditions among MA patients with type 2 diabetes, our findings underscore a significant association between ocular conditions and mental health challenges in older adults, particularly among those with DED disease and glaucoma. In the adjusted models, patients with glaucoma reported significantly more unhealthy mental health days than those with cataracts alone, highlighting the distinct psychological burden associated with this condition. It will be important to further understand this finding in future studies by specifically evaluating the strength of this association with the stage of glaucoma or cataract and the degree of vision loss, if any. Although patients with DED experienced longer durations of poor mental health, the association did not reach statistical significance in the analysis, although the *p*-value was close to the conventional threshold (*p* = 0.06). Given the potential role of DED as an effect modifier, we performed stratified regression analyses by DED status to compare regression results across the two groups.

### 4.1 Ocular conditions, social determinants, and mental health

Among patients without DED, mental health challenges were primarily driven by factors consistent with those observed in the overall population, including higher disease burden (as captured by DCSI), rural residence, and single or other marital status. In this subgroup, lower education, has dual-eligibility status, and male were also associated with a greater number of unhealthy mental health days. However, no ocular condition was statistically significant in this group. In contrast, the subgroup with DED exhibited a distinct risk profile. In this group, absence of dual-eligibility status, female sex, Spanish language, and minoritized race/ethnicity were not protective and, in some cases, were associated with greater mental health problems. The effect size for glaucoma was substantially larger in the DED subgroup than in the total sample [Exp (*β*) = 7.80 vs. Exp (*β*) = 2.69], indicating a stronger association between glaucoma and unhealthy mental health days among patients with coexisting DED. In addition, interaction analyses showed that DED status significantly modified the associations of marital status, education level, and gender with unhealthy mental health days. These findings suggest that patients with DED constitute a clinically distinct population with different psychosocial correlates, and therefore associations observed in the overall may not be directly generalizable to individuals with DED.

While DED has been found to be more prevalent among individuals with glaucoma (21), further work suggests that individuals who have both glaucoma and an ocular surface disease like DED have greater ocular surface symptoms, poorer vision-related quality of life (21), and poorer medication adherence (21). There is a well-known association between glaucoma medications and DED (22, 23). For example, the preservatives used in topical glaucoma medications can be toxic to the eye. Also, some common medications, e.g., prostaglandin analogs, can cause inflammation and irritation of the ocular surface. While glaucoma does not cause DED, the medications commonly used to treat this condition can exacerbate this condition. Work by Tang et al. highlights similar mechanisms affecting mental health between DED and glaucoma, including constant inflammation and dysregulation of sleep as symptoms contributing to depression (24). In addition, the irreversible and progressive nature of vision loss related to glaucoma may lead to continuous mental stress (25). We posit that these factors, compounded with the stress of diabetes management (26) uniquely amplify the prevalence and impact of mental health among patients with both DED and glaucoma.

Patients with lower annual income reported longer durations of unhealthy mental health days. A study by Campbell et al. reported that higher mental and physical HRQOL were associated with both lower medical costs (27). Low-income families with type 2 diabetes face more mental health issues. In addition, our study highlighted that individuals from lower-income households with type 2 diabetes have been reported to experience a greater mental health burden. Many studies have indicated that married individuals have better mental health (28, 29). Living in rural areas is associated with fewer mental health problems, which is consistent with previous study (30). Minority racial groups face greater mental health challenges, whereas White individuals typically have better socioeconomic status, which may contribute to better mental health outcomes. Our findings also suggest that female patients with DED were more likely to report longer unhealthy mental health days compared to their male counterparts. This finding is consistent with work by Kim et al., which found female respondents with DED at greater risks of perceived stress, depression, and anxiety than those without DED (31). Additionally, women with DED have also reported greater frequency and severity of DED symptoms, impact on everyday activities, and greater dissatisfaction with treatment side effects compared to men (32). Given that females have a higher risk of developing DED compared to males (33), these findings further highlight the need for gender-based screening, management, and support strategies in the care of patients with DED. Recognizing the psychosocial dimensions of DED, particularly among women, may improve patient-centered care and mitigate downstream impacts on mental health and quality of life.

Compared to their White counterparts, Black, Hispanic, Asian, and other racial groups with DED reported longer unhealthy mental health days as well. This finding is unique with Zhou et al. reporting race as an insignificant influence in depression for those with DED (34). However as racial and ethnic minorities are associated with worse objective DED parameters and higher odds of having DED (35), they are also less likely to seek, to be referred to, and to utilize tertiary eye care (36), particularly DED care (35). Because Spanish-speaking status and minoritized race/ethnicity were both significantly associated with longer durations of unhealthy mental health days, these factors may reflect overlapping social and structural vulnerabilities among patients with DED. As diabetic racial minority populations have been associated with less access to comprehensive care, worse disease control (37), and a greater risk for depression (38), diabetes-related health access disparities may compound the mental health challenges minority populations with DED face. Our analysis indicates that patients without dual Medicaid and Medicare eligibility were more likely to report longer durations of unhealthy mental health days compared to their dually eligible counterparts. Although most dually eligible patients represent a high-need, high-cost, and vulnerable segment of the population (39), we posit that our findings could potentially reflect disparities in access to comprehensive health and behavioral health services, as dual-eligible individuals often benefit from broader coverage and reduced out-of-pocket costs (40). The lack of dual eligibility may exacerbate financial barriers, limit access to necessary care, and contribute to heightened psychological distress in vulnerable populations.

### 4.2 Limitations and future studies

This study is not without limitations, the identification of DED was based on ICD-10 codes from claims data rather than the Dry Eye-Related Quality of Life Score (DEQ), which may have limited the precision in capturing patient-reported symptom severity and impact (41). Second, the outcome measure of unhealthy mental health days is a general indicator of psychological distress and does not distinguish among specific mental health conditions such as depression, anxiety, or diabetes-related distress. Our findings may be subject to residual confounding due to the lack of information on pre-existing mental health conditions, mental health treatments, psychosocial factors (e.g., social support and stress), and health behaviors (e.g., smoking, alcohol use, physical activity, and sleep). Although we adjusted for multiple clinical and sociodemographic characteristics, these unmeasured factors may have influenced the observed associations between ocular conditions and mental health burden. Missing data were addressed using multiple imputation implemented in PROC MI, with estimates pooled using PROC MIANALYZE; although this approach is widely used, results for variables with substantial missingness should be interpreted with caution due to potential imputation-related bias. For the DED stratified analysis, although diagnostic checks did not indicate undue influence on the model estimates, some observations exhibited relatively high leverage, which may reflect limited covariate overlap in certain subgroups and should be considered when interpreting the magnitude of some estimates. Finally, our sample was restricted to MA beneficiaries aged ≥65 years with type 2 diabetes who completed an HRQOL survey; thus, it may not provide a true representation of the depth of this issue or the broader population. Therefore, caution is warranted when generalizing our findings to all individuals with type 2 diabetes, and further research in more diverse populations is needed.

Future studies should extend the study population beyond MA beneficiaries. Additionally, future research would benefit from examining insurance status for ocular conditions as a distinct independent variable, particularly in light of the fragmented and often limited eye care coverage available to dually eligible beneficiaries (42). Finally, we plan to use longitudinal or time-to-event study designs to better assess temporality and potential causal pathways, with a particular focus on patients with DED and the collection of more detailed data.

## 5 Conclusion

This study adds to the growing body of evidence highlighting disparities in mental health burdens associated with ocular conditions among MA beneficiaries with type 2 diabetes, particularly among patients with DED. Among patients with DED, those with coexisting glaucoma, as well as women, Spanish-speaking beneficiaries, patients identifying as ethnic minority groups, and those without dually eligible patients, reported a higher number of unhealthy mental health days compared to their respective counterparts. In terms of practical significance, our study identifies that patients with type 2 diabetes who also have ocular conditions, particularly those with DED and glaucoma, may experience a greater burden of mental health problems and reduced quality of life. These results suggest that the intersection of specific ocular conditions, chronic disease burden, and sociodemographic vulnerabilities compounds the risk of mental health distress among individuals with diabetes, underscoring the need for integrated, equity-informed, and culturally responsive care models.

## Data Availability

The data analyzed in this study is subject to the following licenses/restrictions: the dataset contains sensitive patient health information and is subject to privacy and confidentiality regulations. Access is restricted and requires approval from the data provider (Humana Inc.) and a signed data use agreement. The data are not publicly available. Requests to access these datasets should be directed to https://developers.humana.com/.
