# Supplementary material for: Mental health burden associated with specific ocular conditions among Medicare Advantage beneficiaries with type 2 diabetes: examining the impact of glaucoma, cataracts, retinopathy, and dry eye on unhealthy mental health days
Source: Front Public Health. 2026 Apr 16;14:1708485. doi: 10.3389/fpubh.2026.1708485 (PMC13128593; doi:10.3389/fpubh.2026.1708485)
Supplement: Supplementary file 1 [file Table_1.docx]

**Supplementary materials**

**Table A.1**

Sensitivity Analysis for the Imputation Method: Complete-Case GLM Analysis

|  | Frequency (%) | Exp(β) | 95%CIs |
| --- | --- | --- | --- |
| Ocular condition  Glaucoma only  Cataracts only  Cataracts with Glaucoma  Others | 90 (15.63)  276 (52.08)  153 (25.56)  50 (8.68) | 2.32 (0.19)***  REF  0.17 (0.45)***  1.13 (0.16) | [1.58, 3.39]  [0.07, 0.42]  [0.82, 1.56] |
| Gender  Female  Male | 227 (39.41)  349 (60.59) | 0.46 (0.17)***  REF | [0.33, 0.64] |
| Age  65–74  75+ | 224 (38.89)  352 (61.11) | REF  1.87 (0.15)*** | [1.38, 2.52] |
| Living in a rural area  No  Yes | 519 (90.10)  57 (9.90) | REF  0.51 (0.24)** | [0.32, 0.82] |
| Annual adult income  ≤ 25,000 USD  > 25,000 USD | 306(53.13)  270 (46.88) | 1.62 (0.16)***  REF | [1.17, 2.25] |
| Education  Less than high school  High school  Above high school | 178 (30.90)  235 (42.53)  153 (26.56) | REF  0.41 (0.16)***  0.39 (0.25)*** | [0.30, 0.56]  [0.24, 0.63] |
| Marital status  Married  Single/others | 300 (52.08)  276 (47.92) | REF  3.15 (0.26)*** | [2.32, 4.27] |
| Primary language  English  Spanish | 564 (97.92)  12 (2.08) | REF  2.37 (0.36)** | [1.16, 4.82] |
| BMI  Below 25 kg/m^2^  25–30 kg/m^2^  Over 30 kg/m^2^ | 5 (0.87)  365 (53.37)  206 (35.76) | 0.00 (244.24)  2.00 (0.18)***  REF | [0.00, NE]  [1.41, 2.83] |
| Race/ethnicity  Non-Hispanic White  Other races | 402 (69.79)  174 (30.21) | REF  2.37 (0.15)*** | [1.77, 3.18] |
| Dry eye  No  Yes | 423 (73.44)  153 (26.56) | REF  2.63 (0.15)*** | [1.94, 3.42] |
| Dual eligibility status  No  Yes | 544 (94.44)  32 (5.56) | 0.44 (0.23)***  REF | [0.29, 0.69] |
|  | Mean (SD) |  |  |
| DCSI | 2.45 (1.89) | 1.09 (0.04)* | [1.02, 1.17] |
| Unhealthy Mental Health Days (>0) | 3.39 (2.39) | -- | -- |

Note: **p* <0.05; ***p* <0.01; *p* <0.001. REF = reference group. SD = standard deviation; NE = not estimated; The value inside the parentheses is the standard error. CIs= Confidence Intervals.

**Table A.2**

Sensitivity Analysis for the Rural vs Non-Rural

|  | Exp(β) | 95%CIs |
| --- | --- | --- |
| Ocular condition  Glaucoma only  Cataracts only  Cataracts with Glaucoma  Others | 2.26 (0.15)***  REF  0.88 (0.14)  1.11 (0.21) | [1.69, 3.02]  [0.65, 1.16]  [0.74, 1.68] |
| Gender  Female  Male | 0.99 (0.11)  REF | [0.80, 1.23] |
| Age  65–74  75+ | REF  0.87 (0.11) | [0.80, 1.08] |
| Living in a rural area  No  Yes | REF  0.67 (0.17)* | [0.48, 0.93] |
| Marital status  Married  Single/others | REF  1.74 (0.13)*** | [1.34, 2.26] |
| Primary language  English  Spanish | 1.55 (0.21)* | [1.02, 2.36] |
| BMI  Below 25 kg/m^2^  25–30 kg/m^2^  Over 30 kg/m^2^ | 0.61 (0.46)  0.99 (0.11)  REF | [0.24, 1.55]  [0.80, 1.23] |
| Race/ethnicity  Non-Hispanic White  Others | REF  1.51 (0.12)*** | [1.21, 1.90] |
| Dry eye  No  Yes | REF  1.23 (0.11) | [0.98, 1.54] |
| Dual eligibility status  No  Yes | 0.92 (0.17)  REF | [0.66, 1.30] |
| DCSI | 1.11 (0.03)*** | [1.06, 1.17] |

Note: **p* <0.05; ***p* <0.01; ****p* <0.001. REF = reference group. The value inside the parentheses is the standard error. CIs= Confidence Intervals.

**Table A.3**

Tests for Interaction Between DED Status and Selected Covariates

| Interaction Term | Exp(β) | 95% CIs |
| --- | --- | --- |
| DED × rural residence | 0.24 (0.49)** | [0.09, 0.62] |
| DED × marital status (single/others) | 0.72 (0.28) | [0.42, 1.23] |
| DED × education (high school) | 6.87 (0.42)*** | [2.97, 15.91] |
| DED × education (above high school) | 15.08 (0.95)* | [1.89, 120.23] |
| DED × female | 7.55 (0.32)*** | [3.96, 14.42] |
| DED × no dual eligibility status | 3.10 (0.41)*** | [1.38, 6.96] |

Note: **p* <0.05; ***p* <0.01; ****p* <0.001. The value inside the parentheses is the standard error. CIs= Confidence Intervals.

**Table A.4**

Part 1: Average Marginal effects (AME) of All Three Pooled Models, Part 2: Adjusted Predicted Mean of All Three Pooled Models

and Part 3: Average Goodness-of-Fit Statistics were Calculated Across the 10 Imputed Models of All Three Pooled Models.

|  | Total sample  (n=992) | | Model 2: Patients Without  DED (n=718) | | Model 2: Patients With DED (n=274) | |
| --- | --- | --- | --- | --- | --- | --- |
| Part 1: AME | | | | | | |
| Contrast | AME | 95%CI | AME | 95%CI | AME | 95%CI |
| Ocular condition  Glaucoma only  Cataracts with glaucoma  Others | 0.98 (0.18)***  -0.16 (0.14)  0.07 (0.21) | [0.61, 1.36]  [-0.45,0.13]  [-0.33, 0.48] | 0.05 (0.42)  -0.19 (0.18)  -0.20 (0.25) | [-0.8, 0.95]  [-0.54, 0.15]  [-0.69, 0.29] | 2.07 (0.28)***  -0.65 (0.61)  -- | [1.53, 2.63]  [-1.86, 0.56]  -- |
| Gender: female | -0.09 (0.12) | [-0.33, 0.14] | -0.45 (0.16)** | [-0.77, -0.12] | 1.28 (0.49)* | [0.27, 2.30] |
| Age: 65–74 | 0.10 (0.11) | [-0.12, 0.32] | 0.16 (0.13) | [-0.10, 0.42] | 0.02 (0.48) | [-1.02, 0.98] |
| Living in a rural area | -0.56 (0.18)* | [-0.89, -0.20] | -0.70 (0.23)* | [-1.15, -0.24] | 0.60 (0.67) | [-0.77, 1.98] |
| Primary language: Spanish | 0.50 (0.21)* | [0.09, 0.92] | -0.21 (0.30) | [-0.81, 0.38] | 2.18 (0.97)* | [0.13, 4.23] |
| BMI  Below 25 kg/m^2^  25–30 kg/m^2^ | 0.55 (0.48)  -0.17 (0.13) | [-0.43, 1.52]  [-0.43, 0.44] | 0.71 (0.56)  -0.27 (0.17) | [-0.43, 1.84]  [-0.62, 0.07] | 0.18 (1.07)  0.43 (0.45) | [-1.97, 2.30]  [-0.53, 1.35] |
| Annual income ≤ 25,000 USD | 0.48 (0.16)** | [0.14, 0.81] | 0.27 (0.35) | [-0.48, 1.02] | 0.36 (0.78) | [-2.04, 1.35] |
| Education  High school  Above high school | -0.21 (0.16)  -0.17 (0.27) | [-0.53, 0.12]  [-0.71, 0.39] | -0.71 (0.26)*  -0.82 (0.64) | [-1.24, -0.17]  [-2.21, 0.59] | 1.04 (0.73)  1.72 (1.67) | [-0.51, 2.61]  [-2.00, 5.40] |
| Marital status: Single/others | 0.64 (0.13)*** | [0.37, 0.90] | 0.73 (0.15)*** | [0.43, 1.03] | -0.88 (0.51) | [-1.98, 0.20] |
| Race/ethnicity/: other races | 0.38 (0.12)** | [0.15, 0.61] | -0.01(0.12) | [-0.27, 0.24] | 3.47 (0.64)*** | [2.19, 4.74] |
| Has no dual eligibility status | -0.11 (0.17) | [-0.42, 0.23] | -0.65 (0.20)** | [-1.04, -0.26] | 1.54 (0.57)* | [0.43, 2.65] |
| Has DED | 0.21 (0.11) | [-0.01, 0.43] | -- | -- | -- | -- |
| DCSI (Continuous) | 0.11 (0.03)*** | [0.06, 0.16] | 0.11 (0.03)*** | [0.13, 0.81] | 0.10 (0.11) | [0.88,1.39] |
| Part 2: Adjusted Predicted Mean | | | | | | |
|  | Total sample | | Patients Without DED | | Patients With DED | |
| Condition | Mean (SD) | 95%CIs | Mean (SD) | 95%CIs | Mean (SD) | 95%CIs |
| Glaucoma-only | 0.74 (0.34)* | [0.06, 1.43] | 0.01 (0.08) | [-0.11, 0.13] | 12.38 (11.82) | [-11.52, 36.28] |
| Cataract-only | 0.42 (0.19)* | [0.04, 0.80] | 0.01 (0.06) | [-0.16, 0.18] | 2.45 (2.19) | [-2.17, 6.80] |
| Part 3: Average Goodness-of-Fit Statistics | | | | | | |
|  | Total sample | | Patients Without DED | | Patients With DED | |
| Scaled Peason χ² | 1.28 | 1.34 | 0.78 | 1.28 | 1.34 | 0.78 |
| Dispersion | 2.44 | 2.22 | 1.39 | 2.44 | 2.22 | 1.39 |
| Power | 1.20 | 1.18 | 1.10 | 1.20 | 1.18 | 1.10 |

Note: **p* <0.05; ***p* <0.01; ****p* <0.001. The value inside the parentheses is the standard error. CIs= Confidence Intervals
